# Development of an RNA Extraction Protocol for Norovirus from Raw Oysters and Detection by qRT-PCR and Droplet-Digital RT-PCR

**DOI:** 10.3390/foods10081804

**Published:** 2021-08-04

**Authors:** Daniel Plante, Julio Alexander Bran Barrera, Maude Lord, Irène Iugovaz, Neda Nasheri

**Affiliations:** 1 Microbiology Laboratory, Regulatory Operations and Enforcement Branch, Health Canada, 1001 St-Laurent Street West, Longueil, QC J4K 1C7, Canada; daniel.plante@canada.ca (D.P.); julioalexander.branbarrera2@canada.ca (J.A.B.B.); maude.lord@canada.ca (M.L.); irene.iugovaz@canada.ca (I.I.); 2 National Food Virology Reference Centre, Bureau of Microbial Hazards, Food Directorate, Health Canada, 251 Sir Frederick Banting Driveway, Ottawa, ON K1A 0K9, Canada; 3 Department of Biochemistry, Microbiology and Immunology, Faculty of Medicine, University of Ottawa, Ottawa, ON K1H 8M5, Canada

**Keywords:** murine norovirus, raw oysters, real-time RT-PCR, droplet-digital RT-PCR, RT-PCR inhibitors

## Abstract

Foodborne viruses such as norovirus and hepatitis A virus cause frequent outbreaks associated with the consumption of raw or undercooked oysters. Viral particles are bioaccumulated in the oyster’s digestive glands, making RNA extraction and RT-PCR detection difficult due to the complex nature of the food matrix and the presence of RT-PCR inhibitors. Herein, we have developed a viral RNA extraction protocol from raw oysters using murine norovirus (MNV) as a surrogate for human noroviruses. The method combines lysis in Tri-Reagent reagent, followed by RNA extraction using Direct-Zol purification columns and lithium chloride precipitation. Viral load quantification was performed by both qRT-PCR and droplet-digital RT-PCR. We have demonstrated that this method can efficiently remove RT-PCR inhibitors, and is sensitive enough to reliably detect viral contamination at 25 PFU/0.2 g. We have also compared the efficiency of this method with the ISO 15216-1:2017 method and Method E developed by Quang and colleagues, and observed significantly higher efficiency compared with the ISO 15216-1 method and comparable efficiency with Method E, with less steps, and shorter hands-on time.

## 1. Introduction

Norovirus is the most common foodborne pathogen, causing 685 million cases of gastroenteritis and approximately 200,000 deaths globally [1]. In 2016, norovirus was responsible for 36% of foodborne outbreaks and 42% foodborne illnesses in the United States [2]. Norovirus infection has led to a total of $4.2 billion in direct health system costs and $60.3 billion in societal costs per year globally [3]. Norovirus is a non-enveloped, positive-sense, single-stranded RNA virus that belongs to *Caliciviridae* family [4]. Based on genetic diversity, norovirus is divided into at least 10 genogroups (G), of which viruses from GI, GII, and GIV infect humans. Noroviruses are further categorized into 60 and 49 different genotypes, based on the sequence diversity in the polymerase and the capsid protein, respectively [5].

Norovirus is also the leading cause of shellfish associated gastroenteritis, and responsible for 48% of all shellfish associated foodborne outbreaks in the U.S. [6]. Frequent norovirus outbreaks have been attributed to the consumption of contaminated raw or undercooked oysters around the world [7,8]. Oysters are particularly considered high-risk for contamination with norovirus because certain oyster species are able to bioaccumulate noroviruses in their digestive tissues due to the presence of human histo-blood group like antigens (HBGA-like antigens) [9,10]. The process of bioaccumulation may even result in contamination of oysters with multiple norovirus genotypes and strains [11].

In 2013, the International Organization for Standardization (ISO) published a two-part technical method for the detection and quantification of noroviruses and hepatitis A virus (HAV) in high-risk food matrices, including bivalve shellfish [12,13], which was later revised to ISO-15216-1:2017 [14]. The ISO-15216-1 method for detection of norovirus from shellfish is based on proteinase K digestion, RNA extraction followed by viral quantification by quantitative reverse transcription PCR (qRT-PCR). This method has been successfully used for norovirus analysis in shellfish by several labs around the world [15,16] and has been adopted for the European baseline survey of norovirus in oysters to estimate the European prevalence of norovirus in raw oysters [17]. However, the extraction efficiency of this method varies significantly with maximum 13.2% for oysters [15,18]. Moreover, various other methods such as direct nucleic acid extraction from digestive glands, virus elution and concentration, with different sensitivity and extraction efficiency have been developed and evaluated [19,20,21]. Recently, Quang Le and colleagues have developed a method for extraction and quantification of norovirus from shellfish based on tri-reagent (Trizol) RNA extraction followed by a purification step using cetyltrimethylammonium bromide (CTAB) treatment and LiCl precipitation [22]. This method provided a significantly higher extraction efficiency compared with the ISO-15216-1 method, and demonstrated that Trizol has superior lysis capability for viral RNA extraction from shellfish [22]. However, the phase separation step and added clean-up steps would make the method arduous and taxing for the analysts and would potentially lead to viral RNA loss due to added steps. Herein, we report the employment of Direct-Zol columns, for RNA extraction directly from oyster tissues lysed with Trizol, without the need for phase separation and precipitation that would potentially lead to RNA loss. We have compared the efficiency of this method with the ISO-15216-1 method as well as the Quang et al. method by applying both qRT-PCR and droplet-digital reverse transcription PCR (ddRT-PCR) for quantification of the viral RNA. We have tested their relative sensitivity in quantifying low and high loads of viral contamination.

## 2. Materials and Methods

### 2.1. Oyster Samples

Raw Malpèque oysters (*Crassostrea virginica*) were purchased at local grocery stores (Longueuil, Québec). Digestive glands were dissected, finely chopped to a paste-like consistency and pooled in petri dishes to ensure the uniformity of the starting material. Portions of 0.2 ± 0.05 g were individually weighed in 2.0 mL microcentrifuge tubes. Portions of 2.0 ± 0.2 g were also prepared for the ISO 15216-1:2017 method used in comparison studies.

### 2.2. Preparation of Murine Norovirus (MNV) Stock

MNV stock were prepared as already described [23]. Briefly, murine BV-2 cells were inoculated with MNV-1 (kindly provided by Dr. Virgin, Washington University School of Medicine, St. Louis, MO, USA). The virus was then extracted after approximately 48 h incubation by 3 cycles of freeze (at −80 °C) and thaw (at room temperature). MNV plaque assay was conducted as described previously [24].

### 2.3. RNA Extractions

Samples were inoculated with varying quantities of MNV suspension at low titer (1.0 × 10^4^ Plaque-Forming Units (PFU)) or high titer (1.0 × 10^6^ PFU) per 0.2 g for protocol development and down to 1 PFU/0.2 g for estimation of the limit of detection. The extraction protocol was divided into lysis, RNA extraction and RNA clean-up steps, with several modifications of the protocol published [22] tested as follows: 

#### 2.3.1. Lysis Followed by Extraction with Direct-Zol Columns

Lysis was done by adding 1 mL of Tri-Reagent (Millipore-Sigma, Mississauga, ON, Canada) to the microcentrifuge tubes containing 0.2 g of oyster tissue. The tubes were vortexed for 30 s and incubated at room temperature for 5 min. The mixture was clarified by centrifugation (13,000× *g*, 1 min, 4 °C) and 800 µL of supernatant were transferred to a clean microcentrifuge tube and mixed with an equal volume of 100% ethanol. The entire volume was processed using Direct-Zol RNA Miniprep Plus columns following manufacturer’s instructions (Zymo-Research, Irvine, CA, USA). Elution was done in 50 µL of nuclease-free water.

#### 2.3.2. Lysis Followed by Extraction with QIAamp Columns

Lysis was done by adding 1 mL of Tri-Reagent to the microcentrifuge tubes containing 0.2 g of oyster tissue. The tubes were vortexed for 30 s and incubated at room temperature for 5 min. Chloroform (250 µL) was added to the mixture and vortexed. Phase separation was done by centrifugation (8000× *g*, 20 min, 4 °C). The aqueous phase (700 µL) was recovered to a clean microcentrifuge tube and mixed with 350 µL of 75% ethanol. The entire volume was processed on QIAamp Viral RNA minikit columns (Qiagen, Mississauga, ON, Canada) as per the manufacturer’s instructions. Elution was done in 50 µL of buffer AVE.

#### 2.3.3. The ISO 15216-1:2017 Method

The extraction protocol described in ISO 15216-1:2017 was followed [14]. Briefly, 2 g portions of homogenized digestive tissue were mixed with 2 mL of proteinase K solution (30 U/mg in nuclease-free water) and incubated for 60 min at 37 °C with agitation (320 rpm), then transferred to a 60 °C water bath for 15 min. Following clarification by centrifugation (3000× *g*, 5 min) the supernatant was transferred to a clean microcentrifuge tube. Two aliquots of 250 µL each were extracted in parallel with the QIAamp Viral RNA minikit (Qiagen, Mississauga, ON, Canada). Final elution was done in 50 µL of nuclease-free water and both extracts were pooled to a final volume of 100 µL.

#### 2.3.4. The Method E 

For Method E by Quang Le et al. [22], 1 g of digestive tissue was mixed in 4 mL Tri-Reagent and sonicated at 30% amplitude, 30 s with a FisherBrand Model 505 sonicator equipped with a 1/8 inch probe (Fisher Scientific, Ottawa, ON, Canada). After 5 min at room temperature, 1 mL of chloroform was added, vortexed and incubated 3 min at room temperature. The mixture was centrifuged (8000× *g*, 20 min, 4 °C). The aqueous upper phase (2.8 mL) was recovered, mixed with 1.4 mL of 75% ethanol and applied to Qiagen QIAamp Viral RNA extraction columns. From there on the manufacturer’s protocol was followed. Final elution was done in 100 µL.

### 2.4. Further RNA Clean-Up

Several protocols were evaluated to further purify the RNA following the Direct-Zol extraction.

#### 2.4.1. Lithium Chloride Precipitation

The eluates were supplemented with 8 M lithium chloride to a final concentration of 2.5 M, stored at −20 °C, 30 min and centrifuged (16,000× *g*, 20 min) to pellet the precipitated material. The pellets were washed with 1 mL of 70% ethanol and air-dried for 30 min. The pellets were dissolved in 50 µL of nuclease-free water and stored at −80 °C.

#### 2.4.2. Cetyltrimethylammonium Bromide (CTAB) and Lithium Chloride Precipitation

The eluates (50 µL) were supplemented with 12.5 µL of 5 M NaCl, 7 µL of CTAB buffer (1 M NaCl, 10% CTAB in nuclease-free water) and 70 µL of a 24:1 mixture of chloroform and isoamyl alcohol. The mixture was briefly vortexed and centrifuged (12,000× *g*, 5 min). The aqueous phase (50 µL) was recovered and precipitated with lithium chloride as described above.

#### 2.4.3. Precipitation in the Presence of Glycogen

The eluates were supplemented with 10 µL of 3 M sodium acetate (ThermoScientific, Nepean, ON, Canada), 2 µL of GlycoBlue Coprecipitant (Invitrogen, Nepean, ON, Canada; 15 mg/mL diluted to 1.5 mg/mL in nuclease-free water) and 120 µL of 100% ethanol. The mixture was briefly vortexed, stored at −20 °C for 30 min and centrifuged (16,000× *g*, 20 min) to pellet the precipitated material. The pellets were washed with 70% ethanol and air-dried for 30 min. The pellets were dissolved in 50 µL of nuclease-free water and stored at −80 °C. 

### 2.5. Detection by qRT-PCR

The qRT-PCR reactions were adapted from Bae and Shwab [25] using the Brilliant II Core RT-PCR reaction kit (Agilent, Mississauga, ON, Canada). Primers MNVKS1, MNVKS2 and probe MNVKS3 were all used at a final concentration of 200 nM. MgCl_2_ was adjusted to a final concentration of 2.5 mM. Two microliters of RNA were used in a final reaction volume of 25 µL. RNA dilution of 1/1 and 1/10 were tested throughout. Reactions were run in a QuantStudio 5 qPCR instrument (Applied Biosystems, Nepean, ON, Canada).

### 2.6. Detection by Droplet Digital PCR

RNA was quantified by ddRT-PCR as described previously [26]. Reactions were run in a QX200 digital PCR system (Bio-Rad, Mississauga, ON, Canada).

### 2.7. Recovery Rates

The recovery rates were calculated with quantitative data obtained by ddRT-PCR. The recovery rate was defined as the ratio of the genome copies recovered from spiked oysters to the genome copies of the MNV used for inoculation [26].

### 2.8. PCR Inhibition

PCR inhibition describes the difference between the expected (Exp.) and measured (Meas.) sample concentration and can be expressed by the following formula: $\mathrm{Inhibition}\;\left(\%\right)\;=100\times \left(\mathrm{Exp}.-\mathrm{Meas}.\right)/\mathrm{Exp}$. We have calculated the level of PCR inhibition in RNA samples extracted by the final protocol by comparing the Ct value obtained from samples tested by qRT-PCR at 1/1 and 1/10 dilutions, taking into account the efficiency of the qRT-PCR reaction. First, we examined the efficiency of the qRT-PCR reaction from the regression analysis of a serial dilution of positive control MNV RNA, with log RNA concentration on the *x*-axis and Ct values on the *y*-axis. The efficiency (E) is related to the slope of the regression line (m) with the following formula: $E=10^{\left(-1/m\right)}-1$ [27]. Efficiency is in turn related to the actual target copy number at each cycle by the formula $\mathrm{Cn}=\mathrm{Ci}\;\times {\left(E+1\right)}^{n}$ [28] where Ci is the number of target molecules at the beginning of cycle n, and Cn is the number of molecules at the end of the same cycle. From these two formulas, it can be shown that a 1-log dilution of the target RNA will delay the Ct value by an amount equivalent to the slope. This simple relationship between dilution, slope and Ct holds true when the RNA is free from PCR inhibitors. The presence of inhibitors delays the Ct value. Diluting the RNA relieves the PCR inhibition so that the diluted sample incurs less or no delay. Consequently, the Ct difference between 1/1 and 1/10 dilutions is less than expected due to the delayed detection of the more concentrated sample. That effect may be used to estimate PCR inhibition. If one considers the concentration of PCR products when fluorescence reaches threshold (Cn), a sample at a given initial concentration amplified under inhibition conditions is equivalent to a sample of a lower concentration amplified for a longer duration in the absence of inhibition. Because Cn is a constant within an experiment, it follows from the above formulas that the concentration of two different samples may be described as $\mathrm{Ci}\;\times {\left(E+1\right)}^{1}=x\;\mathrm{Ci}\;\times {\left(E+1\right)}^{1+∆\mathrm{Ct}}$, where Ci is the concentration of the more concentrated sample. Solving for x gives $x={\left(E+1\right)}^{-∆\mathrm{Ct}}$, which is the ratio of the initial concentration of both samples. Considering that a sample affected by PCR inhibition is functionally equivalent to a sample of a lesser concentration amplified for a longer period, we can assimilate Ci to the concentration of a sample without inhibition and x Ci the apparent concentration of the same sample in the presence on PCR inhibitors and write the PCR inhibition equation as $\mathrm{Inhibition}\;\left(\%\right)=\left(1-{\left(E+1\right)}^{-∆\mathrm{Ct}}\right)\times 100$.

### 2.9. Statistical Analyses

Statistical analyses were performed using the Data Analysis package of Microsoft Excel 2016. The Student’s t-test for equal variance sample sets was used to compare Ct values obtained after two different extraction protocols. When more than two protocols were compared simultaneously, analysis of variance (one-way ANOVA with repetitions) was used, followed by Bonferroni multiple comparison test. The latter is not available in Excel and was done using an online calculator (GraphPad QuickCalcs, GraphPad Software, San Diego, CA, USA).

## 3. Results

### 3.1. Comparing RNA Extraction Efficiency Using Tri-Reagent

**Figure 1:**
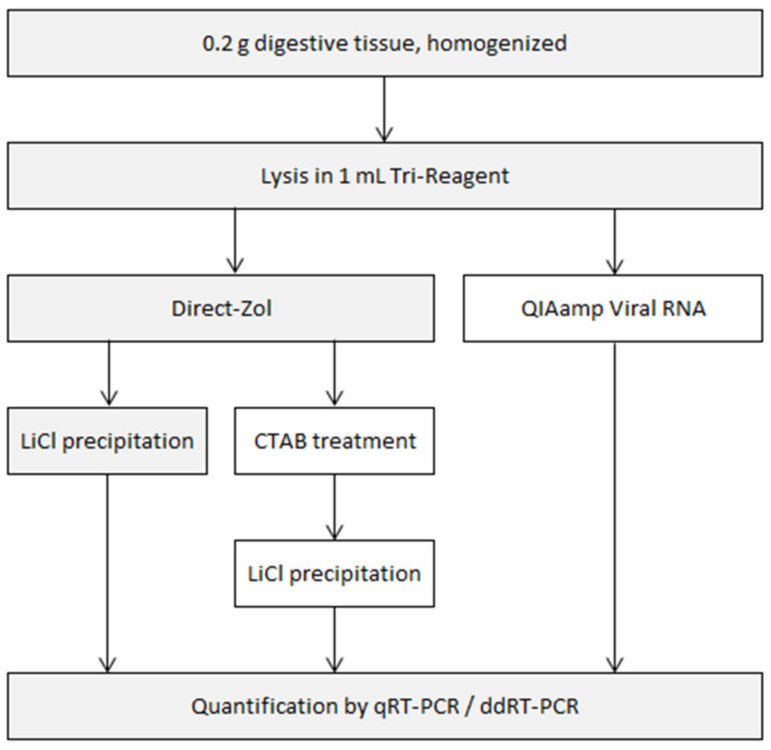
Flowchart of the experimental procedure used in this study. The steps retained in the final extraction protocol are shaded.

Several variations of general RNA extraction and purification protocols were compared. All started with 0.2 g of homogenized digestive tissue lysed in a commercial mixture of phenol and guanidium thiocyanate (Tri-Reagent). RNA was then extracted with one of two silica-based RNA extraction kits. The eluent was used as is, or was further purified as depicted in Figure 1.

**Figure 2:**
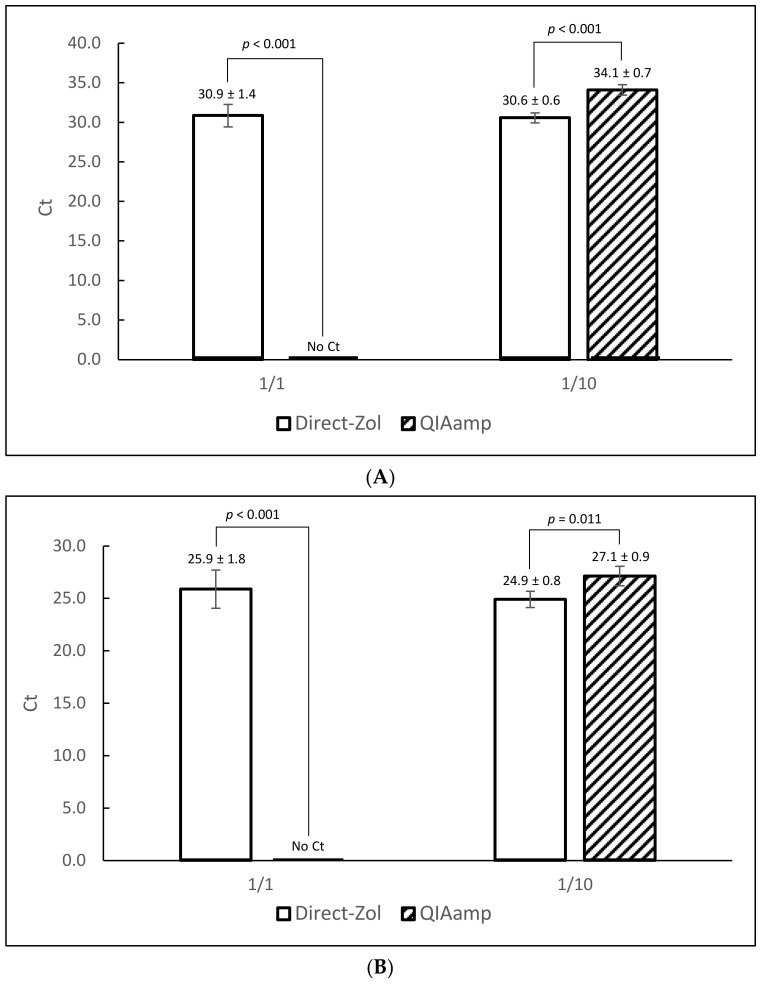
Comparison of Direct-Zol and QIAamp RNA extraction kits at two different MNV concentrations (panel A: 1 × 10^4^ PFU/0.2 g, panel B: 1 × 10^6^ PFU/0.2 g). Each data point represents the average Ct value of 4 extraction replicates with standard deviation. Statistically significant differences (Student’s t test for equal variance sample sets) are shown along with corresponding *p* values. Error bars represent standard deviation. (**A**) low MNV concentration. (**B**) high MNV concentration.

Two RNA purification kits were compared: Direct-Zol RNA Miniprep Plus and QIAamp Viral RNA. The former is designed to work in conjunction with an upstream Tri-Reagent lysis. Following lysis and clarification, the supernatant was mixed with an equal volume of ethanol and applied directly to the affinity column without a need for chloroform extraction. QIAamp Viral RNA is not designed for the same purpose, therefore the Tri-reagent extraction was completed as per manufacturer’s instruction then the aqueous phase was mixed with ethanol and applied to the column. Oyster homogenates were inoculated with two different titers of MNV: 1 × 10^4^ PFU/0.2 g (low) and 1 × 10^6^ PFU/0.2 g (high) in 4 replicates. Extraction efficiency was examined by both protocols and tested by qRT-PCR. Average Ct values of 30.9 ± 1.4 and 30.6 ± 0.6 were obtained from Direct-Zol extractions at 1/1 and 1/10 RNA dilutions, respectively for low titer inoculation (Figure 2A). From QIAamp extractions, positive signals were obtained only after 1/10 dilution of RNA (34.1 ± 0.7). Similar results were obtained with oysters spiked with 1 × 10^6^ PFU/0.2 g (Figure 2B). At that higher level, average Ct values of 25.9 ± 1.8 and 24.9 ± 0.8 were obtained from Direct-Zol extracts at 1/1 and 1/10 dilutions, respectively. QIAamp extracts again were positive only after 1/10 dilution, with an average Ct value of 27.1 ± 0.9. This finding indicates incomplete removal of the RT-PCR inhibitors by QIAamp compared to Direct-Zol, thus we continued the protocol development using Direct-Zol.

### 3.2. Removal of Potential RT-PCR Inhibitors

**Figure 3:**
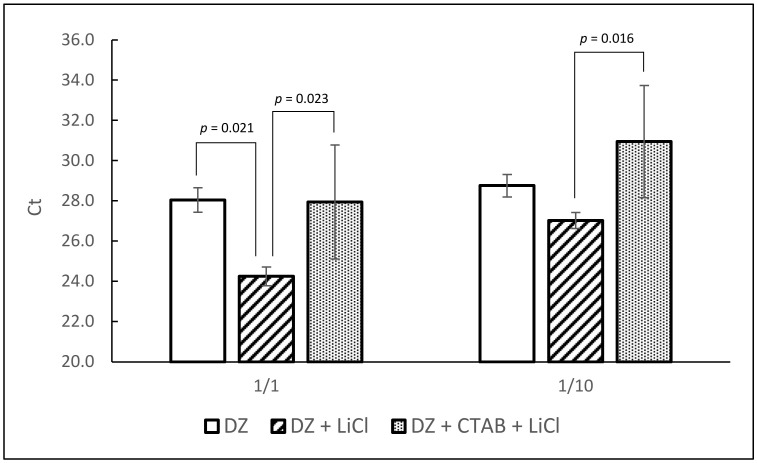
Comparison of three RNA extraction protocol variations (DZ: Direct-Zol only; DZ + LiCl: Direct-Zol followed by lithium chloride precipitation; DZ + CTAB + LiCl: Direct-Zol followed by CTAB precipitation then lithium chloride precipitation). Each bar represents the average of four extraction replicates. Statistically significant differences (ANOVA and Bonferroni multiple comparison tests) are shown with corresponding *p* values. Error bars represent standard deviation.

In order to improve the removal of RT-PCR inhibitors and extraction efficiency, two RNA clean-up protocols were tested: first precipitation of RNA with lithium chloride and second removal of contaminants with CTAB and chloroform isoamyl alcohol followed by lithium chloride precipitation. Extractions were made with homogenized oyster tissue spiked with 1 × 10^6^ PFU/0.2 g in 4 replicates and tested by qRT-PCR. Direct-Zol extractions without further clean-up were done in parallel for comparison. The best average Ct values were obtained after lithium chloride precipitation (Figure 3). As shown, lithium chloride precipitation significantly improved the extraction efficiency compared with Direct-Zol extraction alone. However, treatment with CTAB did not improve the extraction efficiency but increased the standard deviation, probably due to RNA loss because of additional steps. Thus, precipitation with lithium chloride was retained for the rest of the procedure.

### 3.3. Examining the Limit of Detection

**Figure 4:**
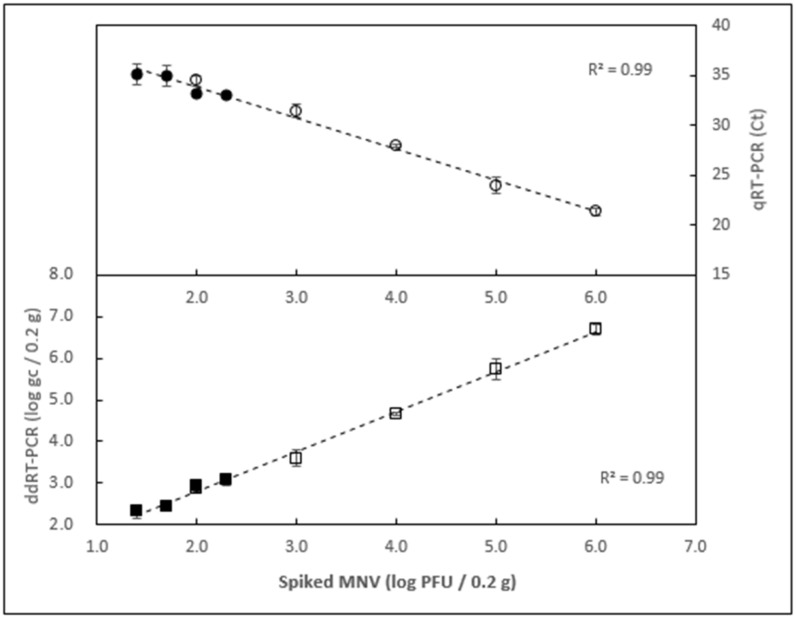
Limit of detection with detection by droplet RT-PCR and qRT-PCR. Samples from two independent experiments (clear markers: 1 × 10^2^ to 1 × 10^6^ PFU/0.2 g; black markers: 2.5 × 10^1^ to 2.0 × 10^2^ PFU/0.2 g) were tested by ddRT-PCR (lower panel) and qRT-PCR (upper panel). Each data point represents the average of three independent extractions. Error bars represent standard deviation.

To measure the limit of detection, homogenized oyster tissue samples were inoculated with a gradient of decreasing amounts of MNV. The range of MNV titer covered from 1.0 × 10^6^ down to 1.0 × 10^1^ PFU/0.2 g of oyster tissue, in two sets. All extractions were done in triplicate and tested by ddRT-PCR. Background level was determined by taking the average signal of all negative controls. As shown in Figure 4 and Table 1, 25 PFU/0.2 g was the lowest dilution that could be reliably detected above the background level for all the replicates (Figure 4), background being defined as the signal from negative control augmented by three standard deviations. At the lowest level tested (1.0 × 10^1^ PFU/0.2 g), only one replicate produced a signal above the background, therefore, this titer was considered below the detection limit. A linear relationship was observed between the viral titer spiked and the amount of genome copies recovered (R^2^ = 0.99) across the entire range tested. The same RNA extracts were also tested by qRT-PCR (Figure 4). Similar to ddRT-PCR results, a spiking level of 25 PFU/0.2 g was the lowest viral load that produced signal above the background for all replicates. A linear relationship was observed between the viral titer spiked and the Ct values recorded (R^2^ = 0.99).

### 3.4. Determining the Extraction Efficiency

The relationship between PFU and genome copies (gc) was calculated with the quantification results of the positive controls (inoculum without oyster tissue) for all spiking levels. The range varied between 7 and 32 gc/PFU, with a weighted average of 28.4 ± 1.0 gc/PFU across all spiking levels (Table 1). Extraction efficiency was obtained by comparing the genome copies recovered from oysters with the genome copies recovered from the corresponding positive controls. The extraction efficiency varied between 16% and 80%, with a weighted average of 21 ± 3% across all spiking levels (Table 1). Interestingly, the recovery efficiency was improved at lower inoculation titers (Figure 4) (Table 1).

### 3.5. Calculation of RT-PCR Inhibition

The efficiency of the qRT-PCR reactions, calculated from a serial dilution of MNV RNA, was found to be equal to 0.948. The slope of the regression line was −3.454. A total of 12 spiked oyster samples from 3 independent experiments, all inoculated at 1 × 10^6^ PFU/0.2 g, were used for the calculations of PCR inhibition. The average Ct of the undiluted samples was 21.97, and that of the 1/10 dilutions of the same samples was 24.77. The average Ct delay was 2.79, which is less than what would be expected if the target RNA was completely free of inhibitors (3.454, equivalent to the slope), indicating the presence of PCR inhibitors. In the absence of inhibition, the expected Ct of undiluted samples would have been 24.77 − 3.45 = 21.32, therefore, the difference between observed and expected was ΔCt = 21.97 − 21.32 = 0.65. From there it follows that the average PCR inhibition of the undiluted samples is $100\times \left(1-{\left(E+1\right)}^{-0.65}\right)=35\%$.

### 3.6. Comparison with Other Extraction Methods

**Figure 5:**
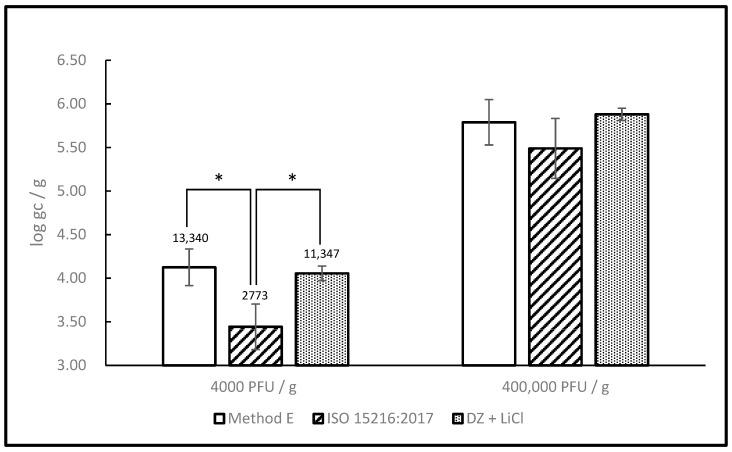
Detection of MNV after extraction with 3 RNA extraction protocols. Oyster digestive gland tissue was inoculated with MNV at 2 different levels (low: 4 × 10^3^ PFU/g, high: 4 × 10^5^ PFU/g) and processed according to the newly developed protocol and two others previously published: Method E and ISO 15216:2017. Significant differences (Bonferroni post-test) are indicated by *. Data represent the average of three independent extractions. Error bars represent standard deviation.

Next, we compared our method with Method E from Quang Le et al. [22] and the ISO 15216-1:2017 method. All three protocols start with the same type of material (homogenized digestive tissue) but the amount is different. Our protocol starts with the least (0.2 g) while ISO 15216-1 starts with the largest amount (2.0 g). Method E falls in-between, with 1.0 g of the starting material. For this reason, inoculation was normalized to a common denominator expressed in terms of PFU/g and the results were expressed in terms of gc/g. Two sets of samples were prepared, one a low spiking level (4 × 10^3^ PFU/g), the other at a higher level (4 × 10^6^ PFU/g). The inoculation and extraction for all three protocols were done simultaneously with the same lot of oysters and inoculum. Our protocol and Method E performed similarly at both spiking levels, however ISO 15216-1 was less sensitive than both, the difference was more significant at the lower titer (Figure 5). 

## 4. Discussion

Here, we have designed an improved protocol for the extraction of norovirus RNA from digestive oyster tissue based on a previously published method [22]. That original protocol, while performing well, is comprised of several steps that make it lengthy and may limit its usefulness for routine analysis. We have made several changes to reduce the complexity of the method while retaining the same sensitivity and robustness. First, the amount of starting material was reduced from 1 g to 0.2 g. That change was made to lower the amount of contaminants that may be dealt with in later stages and possibly allow for a simplified RNA clean-up procedure. While that relatively low amount of starting material may seem to be a potential disadvantage in terms of sensitivity compared to the ISO 15216-1 method, which uses 2 g of digestive tissue, it is notable that in the latter method, the tissue is lysed in 2.0 mL of lysis buffer, but only 500 µL of the clarified supernatant (i.e., 25%) are used for downstream RNA purification. This is equivalent to using approximately 0.5 g of starting material. In our protocol, 0.2 g of digestive tissue is homogenized in 1 mL of Tri Reagent, for which 800 µL (i.e., 80%) are used for RNA extraction. This is equivalent to approximately 0.16 g of starting material. Consequently, even though the ISO 15216-1 method starts with 10 times more material, approximately 3.1 times more digestive tissue is used for RNA extraction. Nonetheless, it is still possible with our method, as with the others, to pool the digestive tissue from several animals to increase the representation of a test portion, provided that homogenization is carried out thoroughly. 

The phenol:guanidium thiocyanate:chloroform extraction of the original protocol was the primary target for simplification. Although proven to yield high-quality RNA, the phase separation step introduces potential RNA loss and the risk of organic solvent carry-over. Moreover, the silica-based RNA extraction kits are not designed to work in conjunction with the aqueous phase of a phenol extraction. Ethanol must be added to the aqueous phase before application to the column but the process is still outside of the manufacturer’s instructions. That is not the case with Direct-Zol RNA extraction kits from Zymo-Research, which, by design, permit the direct application of the TRI-Reagent lysate to the purification column, allowing to by-pass the phase separation step. In comparisons with a more conventional RNA extraction kit (QIAamp Viral RNA minikit), Direct-Zol did perform significantly better, which may be a reflection of the fact that the manufacturer’s instruction of the QIAamp kit were not strictly adhered to and thus QIAamp Viral RNA minikit failed to detect viral RNA in undiluted samples. 

Method E includes two RNA purification steps after elution from the affinity columns: CTAB purification and precipitation in the presence of lithium chloride. We have found that precipitation with lithium chloride leads to a significant improvement in sensitivity as measured in Ct values by qRT-PCR. Purification in the presence of CTAB, however, did not result in any further improvement; in fact, it nullified the gains provided by lithium chloride precipitations and also increased variability. We hypothesize that the combination of a small amount of starting material, combined with the use of an affinity column designed to work with TRI-Reagent lysates, allowed for retention of RNA extracts with relatively small amounts of PCR inhibitors. Consequently, lithium chloride precipitation is sufficient for removing the remaining RT-PCR inhibitors and CTAB treatment resulted in RNA loss without improving RNA purity.

Our final protocol was therefore trimmed down to three distinct steps: lysis in TRI Reagent, extraction using Direct-Zol columns and lithium chloride precipitation. Two steps were removed from Method E: chloroform extraction/phase separation and CTAB purification. The final protocol showed a similar sensitivity but is quicker to perform and generates less toxic waste. 

We have used two different quantification systems; qRT-PCR and ddRT-PCR. Both employed the same PCR primers and fluorescent probes but ddRT-PCR has significant advantages over qRT-PCR. Primarily, ddRT-PCR allows for direct quantification of target RNA without a standard curve [29]. Quantification with qRT-PCR is possible and is routinely done but is prone to several artefacts that need to be controlled and accounted for [30,31]. One major hurdle is that target quantification with qRT-PCR requires a standard curve made out of precisely quantified standards. These standards could be made out of viral RNA or DNA constructs but in each case, two parameters need to be controlled: amplification efficiency of the standards must be the same, or very close to that of the actual samples; and the concentration of the standards must be known with high precision [32]. For norovirus quantification, RNA standards are preferable, but one should consider the risk of degradation of RNA standards over time.

Moreover, the presence of PCR inhibitors can significantly affect the ability of qRT-PCR to provide reliable quantification results. Quantification with ddRT-PCR does not suffer from these drawbacks, as it does not require a standard curve. Results of ddRT-PCR experiments are typically expressed as the number of target copies per microliter of PCR mix, which, with a few simple calculations, can be converted to genome copies per microliter of extracted RNA. 

Furthermore, quantification by ddRT-PCR is less impacted by the presence of inhibitors than qRT-PCR [33,34], because, as long as a positive signal is produced within a droplet (even if the signal is reduced due to inhibitors), the droplet is scored as positive. 

In this study, qRT-PCR was used in the earlier steps of method development, to save on reagents costs. ddRT-PCR was employed later in the study, once a final protocol was generated. The limit of detection of the method was examined using both technologies with the same RNA extracts. There was no obvious difference in sensitivity across the detection platforms, both being able to reliably detect as low as 25 PFU of MNV inoculated into 0.2 g of oyster tissue. The results of ddRT-PCR, however, were fully quantitative, which allowed for precise measurements of the extraction efficiency, defined as the proportion of genome copies inoculated into oyster tissue that were later detected. We first quantified each level of inoculum concentration before comparing the results with those of oyster samples inoculated with the same amount of inoculum. The range of inoculation levels varied between 1 and 1.0 × 10^6^ PFU/0.2 g. We have observed that extraction efficiency varied somewhat across the levels, with values ranging from 16% to 80%. The highest efficiencies tended to be at the lowest spiking levels. We have calculated the average extraction efficiency and, because the range of inoculum levels was very wide, weighted that average based on inoculum concentration and found it to be equal to 21 ± 3%. Extraction efficiency varies widely across different extraction methods, target viruses and shellfish species. For example, efficiencies of 6.4–7.9% (oysters, NoV GI and NoV GII, [35]), 18.5% (oysters, mengovirus, [36]), 15–34% (oysters, HAV, NoV GI, NoV GII and Mengovirus targets, [37]), and 50–85% (oysters, murine norovirus, [6]) have been reported. Therefore, care should be taken when comparing extraction efficiency across different publications. In addition, different shellfish species were shown to respond differently to the same protocol [22]. For that reason, all the experiments reported here were done with only one species of oyster (*Crassostrea virginica*). 

The average level of RT-PCR inhibition of our method is about 35%. This means that the detection of undiluted samples is delayed by less than 1 Ct. Guidelines for PCR inhibition in ISO 15216-1 stipulate that samples are to be tested undiluted if PCR inhibition is less than 75% [14]. The level of PCR inhibition for our method is considerably less. 

Because of the reasons alluded to above, it can be perilous to compare our method with others based on published results only. To allow a fair comparison between methods, we have selected two of them (Quang Le’s Method E, upon which ours is based, and the ISO 15216-1 method, which is currently employed most widely) and used them in parallel with our protocol with the same inoculum and oyster tissue. Doing this, we were able to show that our protocol provides sensitivity similar to Method E but with significantly less hands-on time. Our protocol was also shown to be more sensitive than ISO 15216-1, although the difference was more significant at the lower spiking level, where 11,347 gc/g were recovered with our method as opposed to 2773 gc/g with the ISO 15216-1 method, from identical samples inoculated with the same inoculum. 

In conclusion, we have presented here a new protocol for extraction of norovirus RNA from digestive oyster tissue. The protocol is easy, quick to perform, and yields RNA with only traces of PCR inhibitors. Our data with murine norovirus have shown that it can provide equal or better sensitivity compared to the current gold standard method, ISO 15216-1. Future work will determine if that advantage still holds with human norovirus and other types of shellfish. 

## Figures and Tables

**Table 1:** Calculation of extraction efficiency and relationship between PFU and genome copies. Oyster digestive tissue homogenates were spiked with decreasing concentrations of MNV and extracted. The inocula were also extracted in the absence of oyster tissue for reference. Detection was done by ddRT-PCR. All values are the average of three replicates. Genome copies per PFU and extraction efficiency were calculated for each spiking level; the average of each parameter was weighted according to the target spiking level.

Inoculum (PFU)	Extraction from Inoculum		Extraction from Oysters	
	gc	gc/PFU	gc	Extraction Efficiency
100,000	2,850,900 ± 23,284	29 ± 0.2	627,900 ± 374,279	22%
10,000	285,967 ± 5560	29 ± 0.6	44,773 ± 3878	16%
1000	20,715 ± 552	21 ± 0.6	4232 ± 2103	20%
200	4102 ± 219	21 ± 1.1	1234 ± 386	30%
100	3235 ± 1502	32 ± 15	820 ± 154	25%
50	337 ± 93	7 ± 1.2	268 ± 35	80%
25	345 ± 46	14 ± 1.2	220 ± 91	64%
Weighted Average		28 ± 1.0		21 ± 3%

## Data Availability

The data are contained withing the article.
